# Structural and optical analyses for InGaN-based red micro-LED

**DOI:** 10.1186/s11671-023-03853-1

**Published:** 2023-05-25

**Authors:** Fu-He Hsiao, Wen-Chien Miao, Yu-Heng Hong, Hsin Chiang, I-Hung Ho, Kai-Bo Liang, Daisuke Iida, Chun-Liang Lin, Hyeyoung Ahn, Kazuhiro Ohkawa, Chiao-Yun Chang, Hao-Chung Kuo

**Affiliations:** 1 Semiconductor Research Center, Hon Hai Research Institute, Taipei, 11492 Taiwan; 2 Department of Electrophysics, College of Science, National Yang Ming Chiao Tung University, Hsinchu, 30010 Taiwan; 3 Department of Photonics and Institute of Electro-Optical Engineering, College of Electrical and Computer Engineering, National Yang Ming Chiao Tung University, Hsinchu, 30010 Taiwan; 4 Computer, Electrical and Mathematical Sciences and Engineering (CEMSE) Division, King Abdullah University of Science and Technology (KAUST), Thuwal, 23955 6900 Saudi Arabia; 5 Department of Electrical Engineering, National Taiwan Ocean University, Keelung, 202301 Taiwan

**Keywords:** Micro-LED, Red InGaN-based LED, V-pits, Photoluminescence, Emission efficiency

## Abstract

This study presents a comprehensive analysis of the structural and optical properties of an InGaN-based red micro-LED with a high density of V-shaped pits, offering insights for enhancing emission efficiency. The presence of V-shaped pits is considered advantageous in reducing non-radiative recombination. Furthermore, to systematically investigate the properties of localized states, we conducted temperature-dependent photoluminescence (PL). The results of PL measurements indicate that deep localization in the red double quantum wells can limit carrier escape and improve radiation efficiency. Through a detailed analysis of these results, we extensively investigated the direct impact of epitaxial growth on the efficiency of InGaN red micro-LEDs, thereby laying the foundation for improving efficiency in InGaN-based red micro-LEDs.

## Introduction

Nowadays, light-emitting diodes (LEDs) have become an integral part of our daily lives. In addition to replacing traditional light bulbs, micro-LEDs are a promising platform for visible light communication (VLC) due to their high data transmission rate, fast response, and high efficiency [1–5]. Moreover, micro-LEDs are also seen as potential candidates for next-generation micro-displays [6–8]. There are two main approaches to achieving full-color displays. On the one hand, green and red quantum dots (QDs) or phosphors can be integrated with blue micro-LEDs to function as color converters [9–11]. Nevertheless, there are still several challenges that need to be overcome, such as conversion efficiency, uniformity, and stability. On the other hand, assembling red/green/blue (RGB) LEDs, which are composed of AlGaInP-based red LED and InGaN-based green and blue LED, has been commonly used to achieve full-color displays [12, 13]. While AlGaInP red LEDs demonstrate high efficiencies, their thermal stability is insufficient as LED miniaturization advances. This lack of stability results in elevated operating temperatures of the devices, causing a significant efficiency droop [14–16]. When shrunk size to the micrometer scale, InGaN-based red micro-LEDs not only reduce process costs by using the same material as green and blue micro-LEDs, but also have less of an impact on efficiency due to size reduction and high temperature, thus making them an area of significant interest [17, 18]. However, the development of In-rich and high efficiency for InGaN-based red micro-LEDs is graded as one of the most critical technologies for realizing full-color micro-LEDs displays. For instance, the large lattice mismatch between InN and GaN causes significant strain that decreases the incorporation percentages of indium [19]. This large stress also leads to the degradation of the crystalline quality in InGaN-based micro-LEDs, forming non-radiative recombination centers [20–22]. Moreover, InGaN micro-LEDs with high indium content are subject to quantum confinement, resulting in serious Quantum-Confined Stark effect (QCSE) [20, 21]. The QCSE has a severe impact on the performance of InGaN-based red micro-LEDs, resulting in low external quantum efficiency (EQE) and a significant blue shift in the emission wavelength with increasing injected current [23]. This greatly affects the stability of the red micro-LED in terms of both luminous efficiency and emission wavelength. As a result of containing high indium content, the red micro-LEDs have suffered from low EQE, which has a serious effect on display performance. Therefore, growing high indium content and improving their luminous efficiency have become crucial issues.

Recently, many research groups have been dedicated to developing red InGaN LEDs. Huang et al. [20] were the first to achieve a nitride-based red LED with a light output power of over 1 mW at 20 mA by embedding an AlGaN interlayer on each quantum well (QW). Iida et al. [24] demonstrated a 633 nm red InGaN-based LED by increasing the thickness of GaN buffer layer to reduce the residual in-plane stress. It exhibited an EQE of 1.6% at 20 mA. Chan et al. [25] reported that 633-nm red LEDs had a low forward voltage of 2.25 V with a high active region growth temperature on a relaxed InGaN/GaN superlattice buffer. Ohkawa et al. [26] showed that InGaN-based RGB micro-LED arrays covered 84% of the Rec. 2020 color space. In this work, we demonstrate a high indium content and high-efficiency InGaN-based red LED with increasing the density of V-shaped pits, and we investigate the structural and optical properties of a remarkable epitaxial structure for this InGaN-based red LED. The epitaxial structure with an 8-μm-thick buffer layer was followed from the previous publication by Prof. Ohkawa’s research group [24]. The formation of V-shaped pits plays a critical role in efficiency enhancement. Hangleiter et al. [27] reported in 2005 that V-shaped pits in GaInN/GaN quantum wells can effectively suppress non-radiative recombination, by which to increase the light emission efficiency. It is found that the top of threading dislocations forms the V-shaped pits, which act as energy barriers and effectively hold the carriers from being captured into the non-radiative recombination centers in the InGaN QWs. Thus, the emission efficiency of an InGaN-based LED can be improved [28]. Furthermore, Lee’s research group [29] proposed that the formation of V-pits in GaN-based QWs can also reduce reverse leakage current by electrically passivating dislocations. Therefore, the formation of V-shaped pits plays a critical role in efficiency enhancement.

The indium content of InGaN-based red quantum wells (QWs) can be estimated by X-ray diffraction (XRD) and photoluminescence (PL). Surface morphology was observed using scanning electron microscope (SEM) and atomic force microscope (AFM) measurements. From the PL and time-resolved PL (TRPL) analyses, it is confirmed that a high indium content of red QWs is favorable for achieving longer emission wavelength and deeper localized states. Afterward, the formation of V-pits and the strong localization in QWs are conducive to suppressing non-radiative recombination, resulting in higher radiation efficiency. Based on this structure, we fabricated a red micro-LED device for application in the high-speed visible light communication with an EQE value of 5.0% [30]. Compared to InGaN-based red micro-LEDs, our device demonstrated relatively high efficiency. We aim to explore the reasons for the efficiency improvement in more detail through this article. Therefore, in this study, we investigate the structural and optical properties that contribute to the increased efficiency. The ultimate goal is to further enhance the efficiency of micro-LEDs. We have employed crystal growth conditions similar to those previously published, with an 8-μm-thick buffer layer in Ref. [24]. The growth temperature of the active layer be slightly lowered and then increased the V-pit density. Through this article, we hope to explore the impact of V-pit density on luminous efficiency in depth. This will be a crucial study to understand how epitaxial structures affect micro-LEDs, and it could be believed that the findings from this work will help in the development of high-efficiency red InGaN-based optoelectronic devices.

## Materials and method

**Fig. 1:**
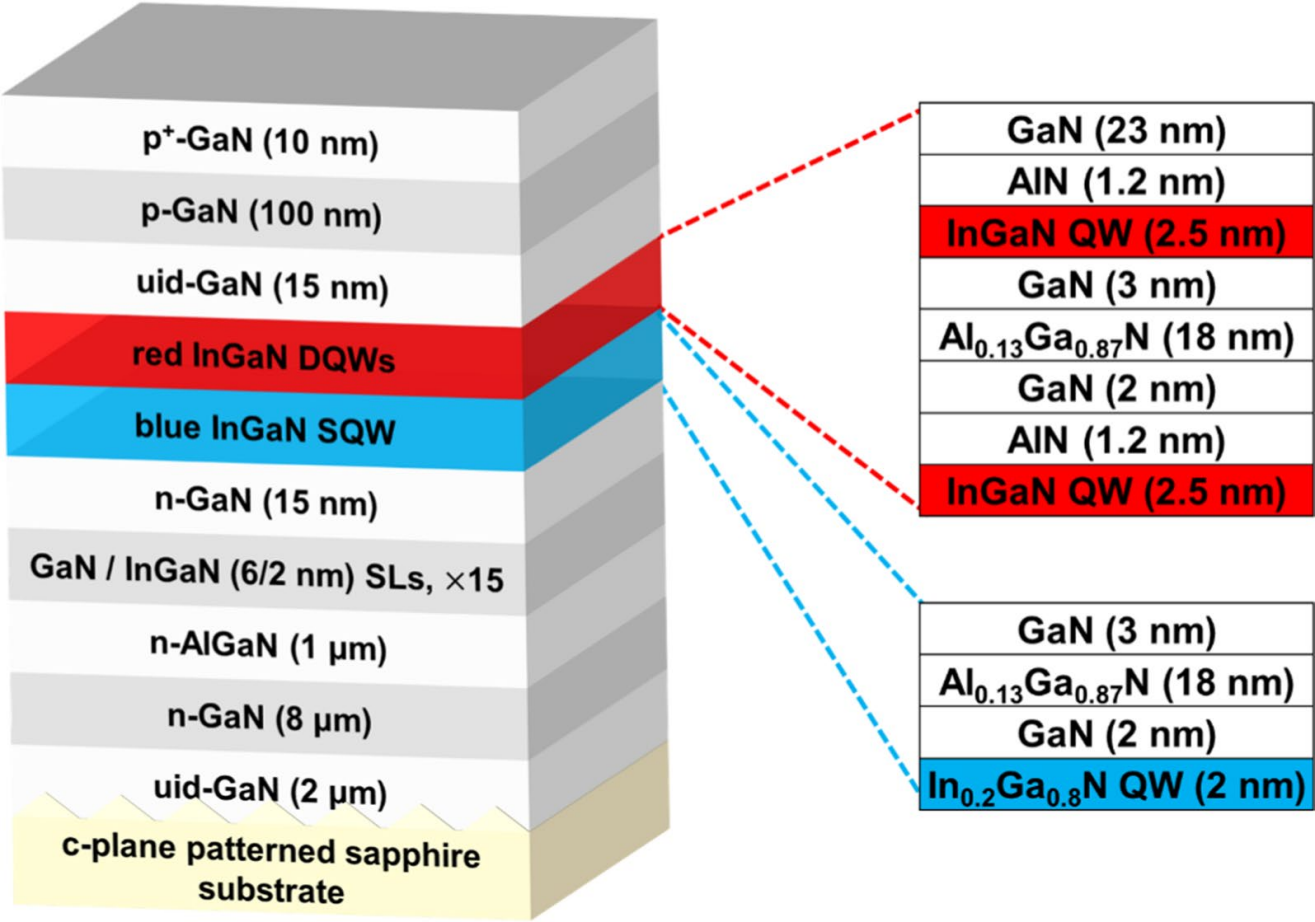
Schematic epitaxial structure of the red InGaN-based LED sample. The upper and lower insets show the detailed structure of the red and blue quantum wells, respectively

A schematic structure of the red InGaN-based LED sample is shown in Fig. 1. The red InGaN-based LED structure was grown via metal–organic vapor-phase epitaxy (MOVPE) on a *c*-plane patterned sapphire substrate (PSS). The epitaxial process started with a 2-μm unintentionally doped (uid) GaN layer, followed by an 8-μm-thick *n*-GaN buffer layer, 1 μm *n*-AlGaN, and 15 pairs of GaN/InGaN superlattice (SL) layers. Afterward, a 15-nm *n*-GaN, low-In-content blue InGaN single QW (SQW), and high-In-content red InGaN double QWs (DQWs) were grown as the active regions. The insets in Fig. 1 show the detailed structure of the blue and red QWs. For the blue SQW structure, it consists of In_0.2_Ga_0.8_N QW and GaN/Al_0.13_Ga_0.87_N/GaN barrier layers. The blue SQW contributes to reduce strain and improve the optical properties of InGaN-based red LEDs [31]. As for the red DQWs structure, it is composed of In_0.34_Ga_0.66_N DQWs and AlN/GaN/Al_0.13_Ga_0.87_N/GaN barrier layers, while GaN was used to replace the Al_0.13_Ga_0.87_N part in the upper barrier. The base growth recipe was identical to that of Ref. [24]. However, the growth temperature for the red QWs was slightly lower than that used for regular red LED growth. Finally, a 15-nm undoped GaN, 100-nm *p*-GaN, and 10-nm *p*^+^-GaN contact layer were grown [30].

The quality and morphology of InGaN epitaxial structure were examined using high-resolution XRD, scanning electron microscope (SEM), and atomic force microscope (AFM). All PL measurements, including power- and temperature-dependent PL, as well as temperature-dependent TRPL, were conducted, while the sample was mounted in a helium closed-circuit cryostat, and the temperature was precisely controlled between 20 and 295 K. The excitation source was a pulse laser with a wavelength of 405 nm and a repetition rate of 10 MHz, and the laser spot diameter was 8 μm.

## Results and discussion

### Structural properties of epitaxial structure

#### Surface morphology

**Fig. 2:**
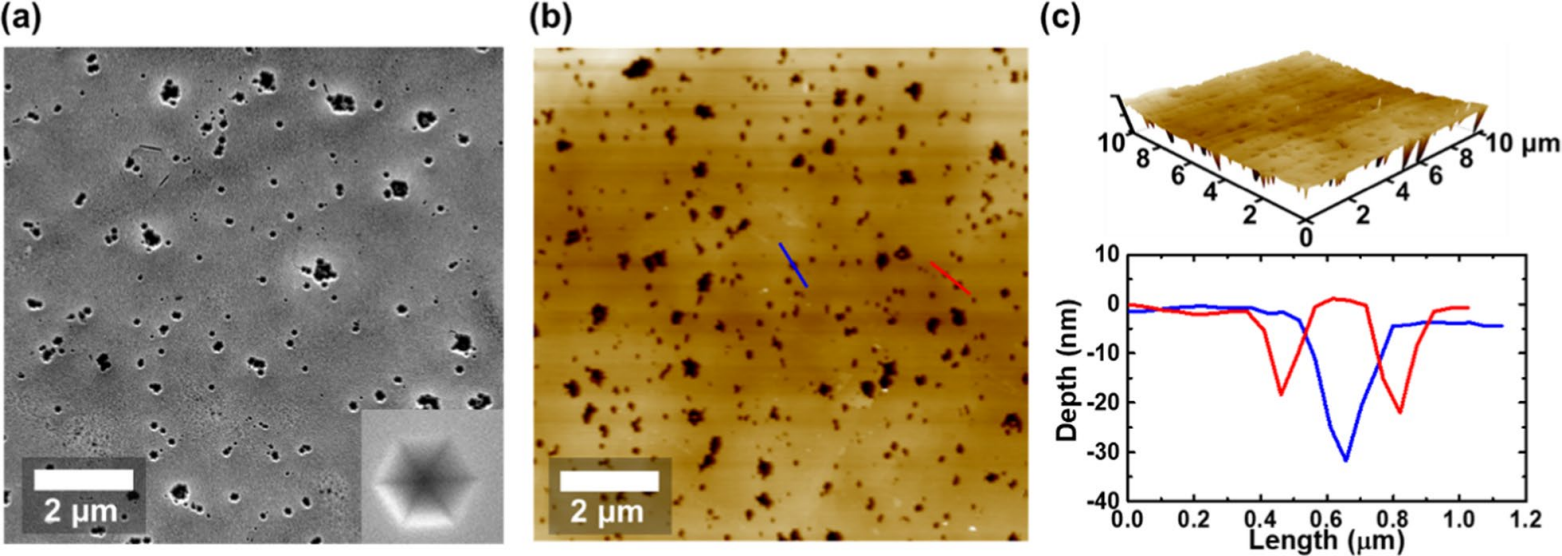
Morphology of red InGaN-based LED surface. **a** SEM image of the surface, with the inset showing the V-pits at higher magnification. **b** 2D AFM images of the surface. **c** Upper: 3D AFM image of the surface for the full area of figure **b**. Lower: the section profile of the blue and red cut line in figure **b**

To investigate the surface morphology of our sample, we used SEM and AFM as the most suitable methods. The SEM and AFM images are shown in Fig. 2. Both exhibit numerous black spots on the sample surface. The section profile and the 3D AFM image provide evidence that the black spots are pits on the surface, with the small spots being V-shaped pits, whereas the large spots are either trench defects or the aggregation of multiple V-shaped pits [24, 32]. The formation of the trench defects was attributed to basal stacking faults in red DQWs, which resulted in decreased internal quantum efficiency (IQE) of the active region [24, 33]. On the flip side, V-shaped pits were formed in the InGaN/GaN SLs in the structure. The origin of V-shaped pits in the sample was confirmed through a cross-sectional transmission electron microscopy (TEM) image in our previous publication [24]. Threading dislocations and other structural defects arose from the lattice mismatch and thermal expansion coefficient differences between the GaN and the substrate. The V-shaped pits were found to originate at threading dislocations and extend along the *c*-axis growth direction in the InGaN epilayer. Thus, the InGaN QWs were disrupted through the formation of inverted hexagonal V-shaped pits [34, 35].

The inset in Fig. 2a confirms the formation of the hexagonal V-shaped pits on the InGaN QWs. It should be noted that the growth temperature for the red QWs in this structure was lower than that used for regular red LED growth. In addition, some groups have reported that the dominant factor for the formation of V-pits appears to be low-temperature growth [36, 37]. Therefore, a higher V-pit density was expected in the sample due to the high In composition in the red QWs, which is likely to increase surface defects. The V-pit density in our sample is directly estimated to be 3.23 × 10^8^ cm^−2^ from Fig. 2a, while the V-pit size of about 140–180 nm is observed. The QWs cover the V-shaped pits, forming a narrower QW thickness on the sidewalls of the pits. The QWs on the inclined V-shaped pits have a higher energy barrier, which effectively prevents carriers from entering threading dislocations, thus shielding the non-radiative recombination centers [24, 28]. Therefore, the high density of V-shaped pits is beneficial for the performance of optical properties. Besides, the formation of the V-shaped pits may cause strain relaxation in the InGaN/GaN epilayer, improving the crystalline quality, resulting in more indium content and a longer emission wavelength [38].

#### High-resolution X-ray diffraction

**Fig. 3:**
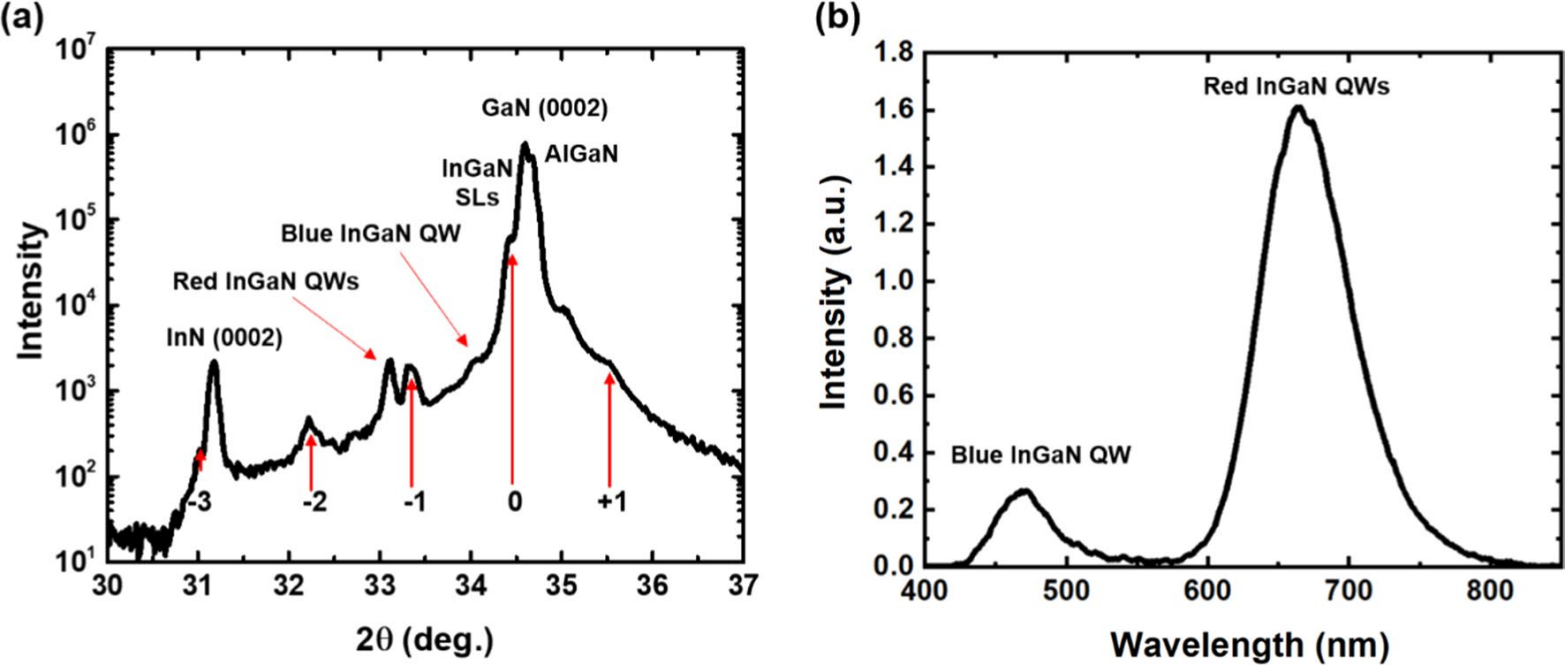
**a** X-ray $\theta$–2$\theta$ diffraction patterns of the sample. The peaks corresponding to the In_*x*_Ga_1−*x*_N are shown. **b** PL spectrum at room temperature with low excitation fluence

To obtain further information about the epitaxial structures, we performed XRD measurements to analyze the sample's structure. Figure 3a illustrates $\theta$–2$\theta$ XRD patterns of the sample at room temperature (300 K). The strongest peak observed at 2$\theta$ = 34.60°, corresponding to GaN (0002), mainly originates from the cap and barrier layers. The peak at 2$\theta$ = 32.18°, corresponding to the indium-rich phase, is identified as InN (0002) [39]. The extended peak at 2$\theta$ = 34.66° is recognized as the AlGaN barrier layer in the QW structure. The adjacent peak on the left shoulder of the GaN peak is the zero-order peak of the GaN/ In_0.08_Ga_0.92_N SL structure. Higher-order (− 3rd to 1st) satellite peaks can be observed on either side of the zero-order SL peak. The average period length of the SL can be calculated based on equation [40]:

1 $$L_{{{\text{ave}}}} = \frac{n\lambda }{2}\left( {\sin \theta_{n} - \sin \theta_{{{\text{SL}}}} } \right)$$

where *n* is the order of the satellite peak; $\theta_{n}$ is the diffraction angle at the *n*th-order peak; $\theta_{{{\text{SL}}}}$ is the angle of the zero-order peak. The average length of the period obtained from XRD patterns is 8.35 nm. The result can be confirmed by Zhuang et al. [41] that cross-sectional STEM image of the sample shows an 8.3-nm-length period of the SL structure.

As for the peak of the hybrid InGaN QWs, we cannot distinguish the individual peak directly from the XRD due to the fringe peak originated from the AlGaN and GaN. The zero-order hybrid InGaN QWs are either hidden or nearby the underlying layer and difficult to analyze. However, the 8-μm-thick underlying GaN buffer layers are beneficial in reducing the residual in-plane stress [24]. Besides, Iida et al. [31, 42] demonstrated that the introduction of Al atoms in the barrier layers and blue SQW are expected to provide strain compensation and enhance the optical properties of red InGaN DQWs. Therefore, it suggests that the red InGaN DQWs would be growth in better crystalline quality. Furthermore, increasing indium incorporation in the red In_*x*_Ga_1−*x*_N DQWs by taking advantage of strain relaxation could be considered [43]. We observed blue and red PL emission at room temperature with low excitation fluence as shown in Fig. 3b. The blue emission peak at 470.18 nm is assigned to the InGaN single QW, and the high-In-content red emission peak at 669.27 nm is attributed to the InGaN DQWs. Based on the XRD measurement and the PL result, the estimation of the indium content, namely the x of indium incorporation in the red In_x_Ga_1-x_N DQWs could be roughly estimated to be approximate 0.34. In the next section, we will take a deep dive into the more detailed optical analyses of the high indium content InGaN epitaxial structure.

### Optical properties

In this section, we will analyze the optical properties, localization behavior, and thermal quench of red In_*x*_Ga_1−*x*_N DQWs through PL measurements. Specifically, we will focus on the red light emission and select a PL spectrum range from 515 to 775 nm. The profitable influence of strain relaxation with high indium content on the optical properties is explored and discussed.

#### Power-dependent PL

**Fig. 4:**
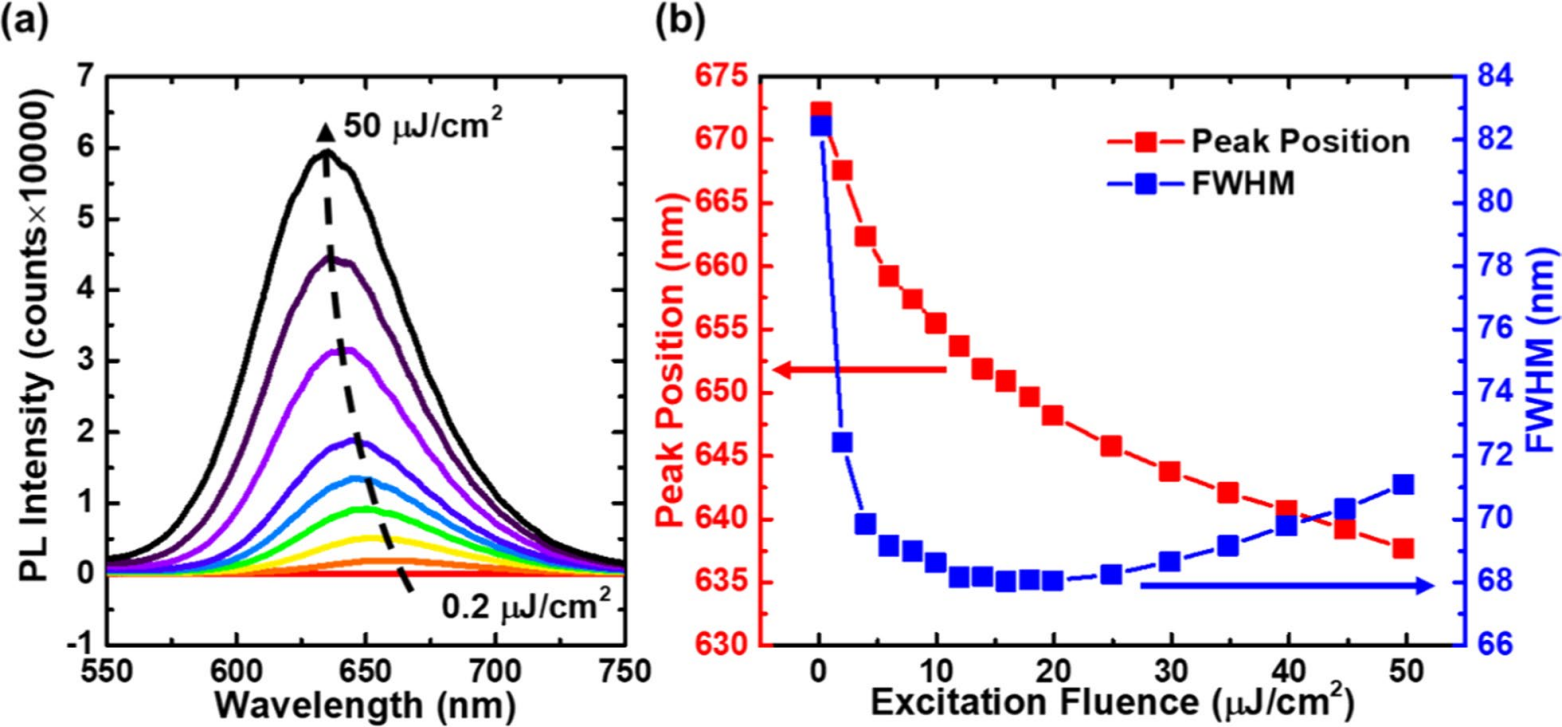
Power-dependent PL spectra. **a** PL spectra of the sample for various excitation fluences at room temperature. **b** Emission peak position and full width at half maximum (FWHM) as functions of excitation fluence at room temperature

Figure 4 depicts the power-dependent PL spectra at room temperature (295 K). As shown in Fig. 4a, the PL intensities exhibit a significant enhancement as the excitation fluence increases from 0.2 to 50 μJ/cm^2^. The peak shift and full width at half maximum (FWHM) variation as functions of excitation fluence are presented in detail in Fig. 4b. The peak position exhibits a monotonic blueshift from 672.2 to 637.7 nm with increasing excitation fluence, while the linewidth initially decreases in the low excitation range and drops to the minimum value of 66 nm. After the minimum value, the FWHM starts to broaden again as the excitation fluence further increases. Such a long wavelength and wide FWHM suggest that there is indeed a high indium content in the red DQWs.

There are two possible reasons for the observed blueshift in the PL peak. One explanation refers to the screening of the piezoelectric field, and another is related to band-filling in the localized energy states in InGaN QWs [44–46]. As the excitation fluence increases, the piezoelectric fields in the strained InGaN well layers are screened by the internal electric fields, which leads to the blueshift in the emission PL peak. In addition, with a further increase in the injected carrier density, the band-filling effect of highly energetic localized centers also takes part in the emission process and becomes dominant. With regard to the PL linewidth, the observed narrowing and broadening can be explained by the Coulomb screening of the QCSE and the band-filling effect [44, 46]. It has been found that the excitation fluence value representing a transition between the screening effect and band-filling effect is approximately 15 μJ/cm^2^ at room temperature.

#### Temperature-dependent PL

**Fig. 5:**
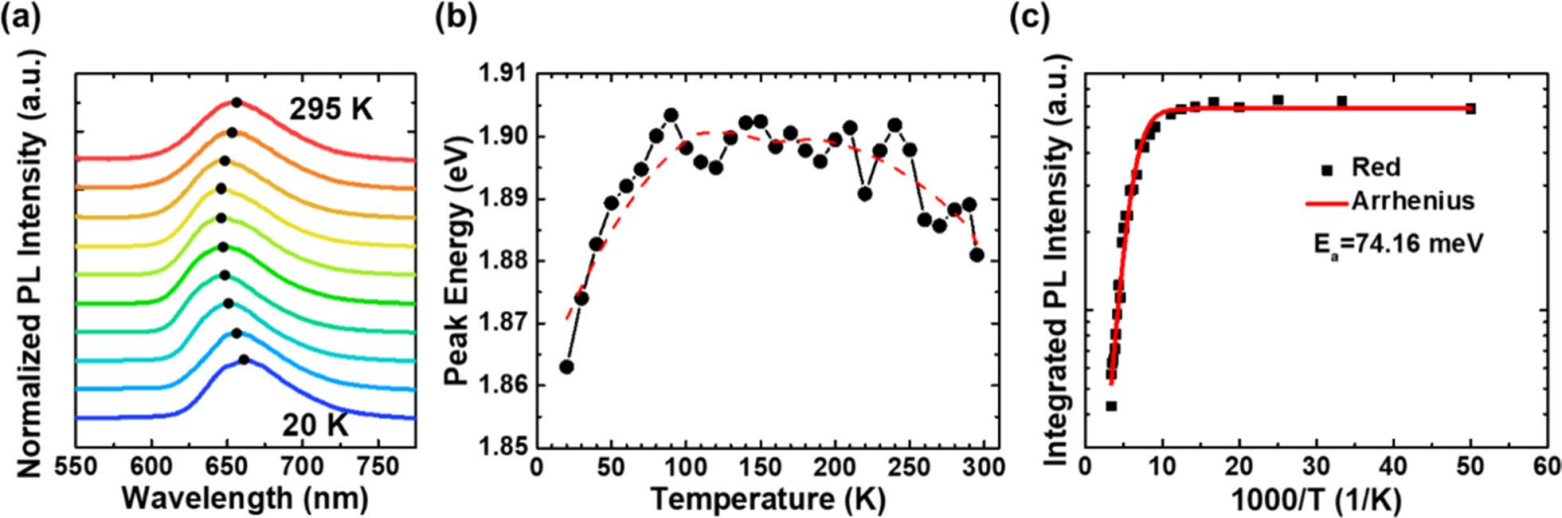
Temperature-dependent PL spectra. **a** The normalized temperature-dependent PL spectra with 20–295 K. The black dots represent the PL peak position at various temperatures. **b** Temperature dependence of PL peaks energies at excitation fluence ~ 6 μJ/cm^2^. **c** Arrhenius plot of PL intensity versus temperature at excitation fluence ~ 6 μJ/cm^2^. The red line is the fitting curve using the Arrhenius model

After comprehending the optical behavior at room temperature, we proceeded to investigate the optical properties and carrier dynamics at different temperatures for further analysis. Figure 5a displays the results of temperature-dependent PL of samples ranging from 20 to 295 K at weak excitation fluence ~ 6 μJ/cm^2^. We selected a small excitation fluence that is less than 15 μJ/cm^2^ to avoid band-filling effects at the beginning of the measurement. The spectra were normalized and vertically shifted for better observation. The black dots in the figure depict the PL peak position at various temperatures. Surprisingly, no S-shaped temperature-dependent behavior of the luminescence peak energy was observed, which is a characteristic feature of InGaN QWs and has been reported by many groups [47–49].

After analyzing the emission properties at different temperatures, we further clarified the detailed peak energy positions in Fig. 5b. Though the PL peak energies seem to fluctuate due to some measurement or fitting errors, the trend showed a blueshift at low temperature, followed by a slight redshift with increasing temperature. Imperfections in the QW structure, such as deep states formed by fluctuations of indium component inside well layers and shallow states formed by fluctuations of well thickness, are available to capture free carriers to form localized carriers. It has been reported that a high excitation power could result in the absence of S-shaped behavior [46]. However, our study may not be in the same situation, as we conducted temperature-dependent PL measurements under a weak excitation fluence of ~ 6 μJ/cm^2^. In contrast to the S-shaped temperature-dependent behavior, we did not observe any redshift in our experiment at low temperatures. This suggests that carriers are strongly localized due to the localized states in red QWs with high In content [50–52].

**Fig. 6:**
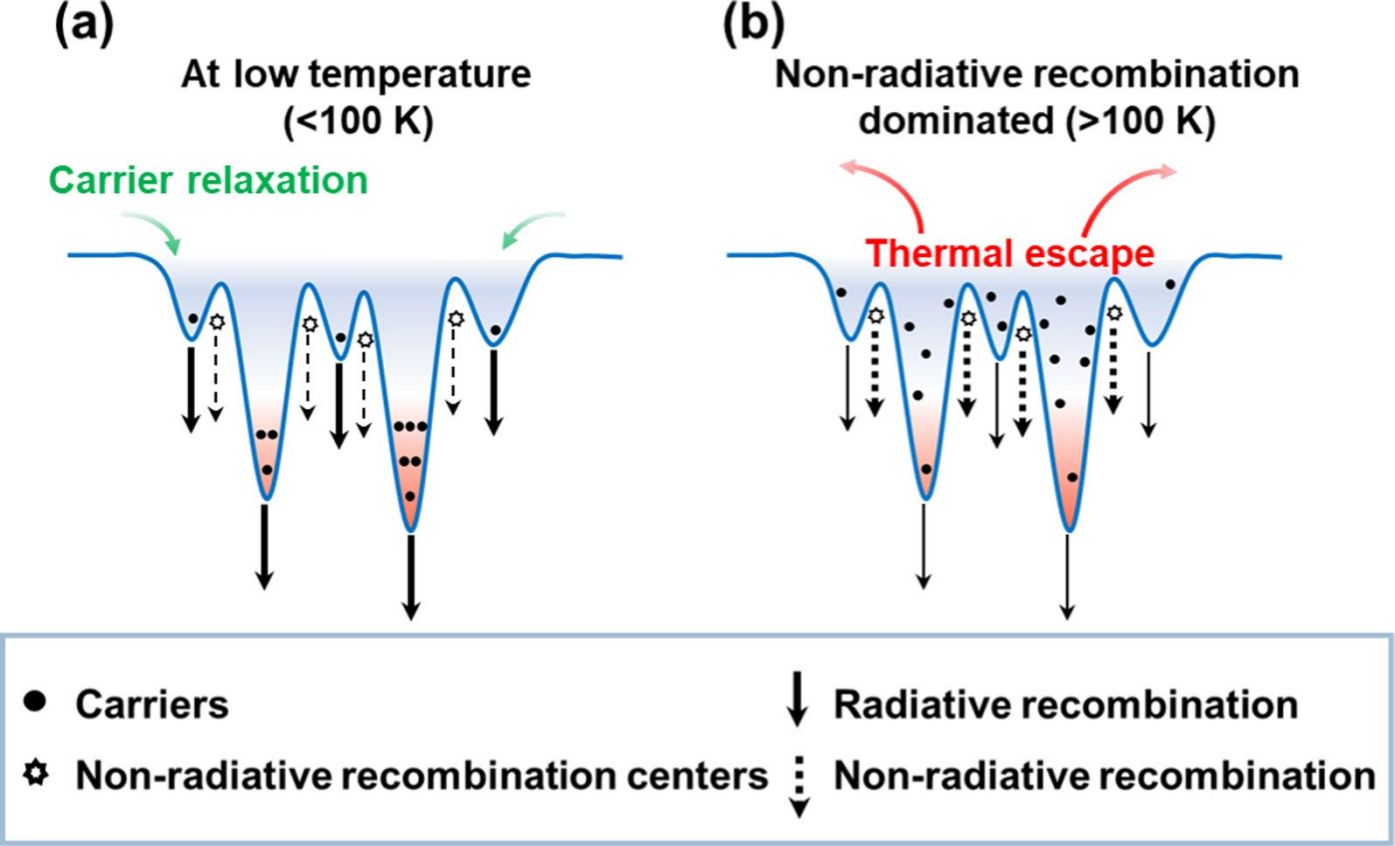
Schematic diagrams indicating the possible mechanism of carrier dynamic in the red QWs structure below and above 100 K. **a** The carrier distribution at temperature lower than 100 K. **b** The carrier distribution at temperature higher than 100 K

To provide a detailed explanation of the temperature-dependent behavior of the peak energy observed in our study, we present schematic diagrams in Fig. 6 that indicates the possible carrier dynamics in the red QWs structure at different temperatures with excitation fluence ~ 6 μJ/cm^2^. At low temperatures, carriers are randomly distributed among deep and shallow potential minima. When the temperature (*T*) increases, in the range of *T* < 100 K, the confinement energy in localized states is very large, so the thermal activation cannot make the carriers transfer from shallow states to deeper states via hopping [46, 50]. Therefore, the carriers remain in the localized states where they were initially distributed. In contrast, as the temperature increases, the carriers achieve thermal equilibrium with the lattice and occupy higher energy levels of the localized states, resulting in a blueshift of the peak energy. For *T* > 100 K, the non-radiative dislocation process tends to dominate, which means that carriers start to escape from the localized states. As a result, fewer carriers undergo radiative recombination process in the localized states. The tendency of the peak energy redshift is then observed due to the temperature-induced bandgap shrinkage [46, 52, 53].

To further explore the thermal quenching effect of photoluminescence in the red active region, we plotted the integrated PL intensities of the red emission as a function of the reciprocal of temperature in Fig. 5c. Assuming the integrated PL intensity at low temperature (20 K) is 100%, we found that the integrated PL intensity of the sample at ambient temperature is only 7.26%. As the temperature increases above 100 K, the PL intensities show a significant drop. The thermal quenching of PL intensities is attributed to non-radiative recombination, which gradually dominates the recombination process at high temperatures.

To determine the thermal activation energy, we employed the one-channel Arrhenius model to analyze the temperature-dependent PL intensities of the sample in the range from low (20 K) to ambient temperature [43, 47, 48, 50]. The equation of this model is as follows:

2 $$I\left( T \right) = \frac{{I_{0} }}{{\left[ {1 + A \cdot \exp \left( { - \frac{{E_{a} }}{{k_{{\text{B}}} \cdot T}}} \right)} \right]}}$$

where *I*_0_ is the integrated PL intensity at low temperature, *A* is the constant related to the density of non-radiative recombination centers, *E*_a_ is the activation energy of the corresponding non-radiative centers induced by defects, and *k*_B_ is the Boltzmann constant. The Arrhenius model fitting curve is presented in Fig. 5c, revealing an *E*_a_ value of 74.16 meV. This value is comparatively higher than the value reported for blue and green LEDs [54, 55]. Activation energy is usually attributed with the carrier confinement capability within potential minima. Therefore, it can be inferred that the significant carrier localization in the sample results in strong confinement, which aligns with the absence of S-shaped behavior.

At lower temperatures, localized states can easily capture and trap carriers due to non-radiative recombination centers being frozen and inactive, as illustrated in Fig. 6a. On the contrary, at higher temperatures, both the carriers in localized states and non-radiative centers are thermally activated [50]. This thermally activation causes carriers to escape from the localized states, as shown in Fig. 6b. Subsequently, the delocalized carriers are then trapped by the non-radiative centers, resulting in the non-radiative recombination process. As a result, the quenching PL intensity comes into sight at high temperatures.

#### Temperature-dependent TRPL

**Fig. 7:**
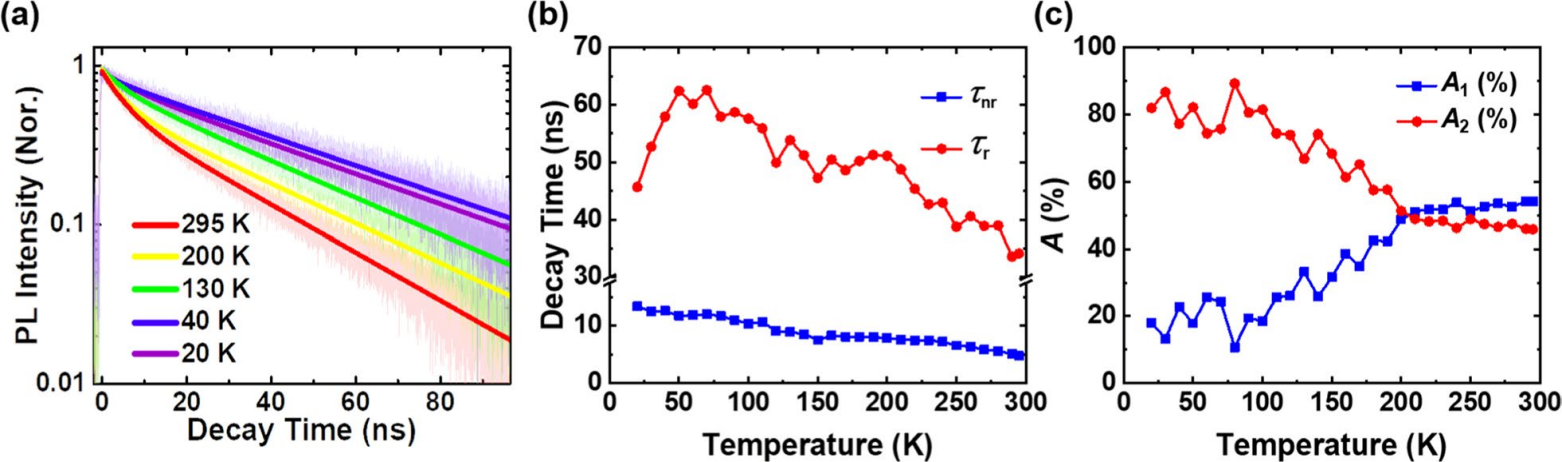
Temperature-dependent TRPL spectra and fitting parameters. **a** TRPL decay spectra at various temperatures. The translucent backgrounds are the raw data. The solid lines are the fitting curve of the raw data with a bi-exponential decay function. **b** Temperature dependence of the decay times derived from bi-exponential decay function. **c** Temperature dependence of the ratio of *A*_1_ and *A*_2_

To obtain more detailed information on the dynamics of carriers in red InGaN DQWs, we measured temperature-dependent TRPL curves of this sample at various temperatures. Normalized temperature-dependent TRPL spectra are shown in Fig. 7a. The TRPL curves are translucent and are shown in the figure. The TRPL decay curves can be well-fitted with a bi-exponential function as shown in Fig. 7a. The equation is as follows:

3 $$I\left( t \right) = A_{1} \times \exp \left( {\frac{ - t}{{\tau_{{{\text{nr}}}} }}} \right) + A_{2} \times \exp \left( {\frac{ - t}{{\tau_{{{\text{r}}}} }}} \right)$$

where parameters $\tau_{{{\text{nr}}}}$ and $\tau_{{\text{r}}}$ represent the non-radiative and radiative lifetimes, respectively, and *I*(*t*) is the TRPL intensity at time *t*. The intensities of the recombination process are determined by *A*_1_ and *A*_2_, which are correlated with the number of carriers captured by the non-radiative and radiative channels, respectively.

As illustrated in Fig. 7a, the decay rate of the sample initially slows down slightly (form 20 to 40 K), then turns faster (from 40 to 295 K) with raising the temperature. A more detailed temperature-dependent decay time derived from a bi-exponential function is revealed in Fig. 7b. It is well-known that the carrier recombination process of InGaN-based micro-LEDs is dominated by radiative recombination at low temperature (20 K). From Fig. 7c, it can be seen that the *A*_2_ coefficient dominates the transition ratio at low temperature 20 K, through which we can judge that the slower lifetime may be attributed to radiative recombination. The higher transition rate of non-radiative recombination compared to radiative recombination may be the cause of the current high defect density. And it can be observed that $\tau_{{{\text{nr}}}}$ gradually decreases as the temperature grows. Nonetheless, $\tau_{{\text{r}}}$ initially increases from 45 to 65 ns, and then decreases to 35 ns afterward as the temperature grows. In fact, the decay time increasing indicates that the radiative recombination process is more dominant and occurs in certain localized states in the temperature range below about 70 K [55, 56]. For *T* > 100 K, the decay lifetime decreases from 55 ns, indicating that non-radiative recombination starts to be activated and hangs over the recombination process at higher temperatures. Interestingly, this result is consistent with the findings of the temperature-dependent PL, which both of them suggest that the non-radiative recombination begins to intensify when the temperature is greater than 100 K. Hence, we can consider that *T* = 100 K as the critical temperature of the activation of the non-radiative recombination process in our measurement. By combining the efficiency and the decay time of TRPL, the relationship between measured PL decay time $\left( \tau \right)$, $\tau_{{\text{r}}}$, and $\tau_{{{\text{nr}}}}$ can be described as [43, 51, 54, 57]:

4 $$\frac{1}{\tau } = \frac{1}{{\tau_{{\text{r}}} }} + \frac{1}{{\tau_{{{\text{nr}}}} }}$$

5 $$\tau_{{\text{r}}} = \tau \times \frac{1}{\eta }$$

6 $$\tau_{{{\text{nr}}}} = \tau \times \frac{1}{1 - \eta }$$

where the variable $\eta$ denotes the temperature-dependent IQE, which can be computed via dividing the PL decay time by the radiative lifetime at arbitrary temperature. It is found that the IQE of the sample at ambient temperature is about 12.22%.

It is also worth noting that the coefficients $A_{1}$ and $A_{2}$, which are associated with the ratio of the number of carriers captured by non-radiative and radiative channels, exhibit variability across different temperatures, as shown in Fig. 7c. The figure indicates that the ratio remains relatively stable, around 80%, at temperatures below the critical temperature. However, with the activation of the non-radiative recombination process at temperatures higher than 100 K, more and more carriers escape from the localized states due to thermal effects, resulting in fewer localized carriers participating in the radiative recombination process. As the result, the ratio of *A*_1_ and *A*_2_ is getting closer and closer, and *A*_1_ surpasses *A*_2_ at 200 K. It reveals that the non-radiative recombination evidently dominates at temperatures higher than 200 K; however, the ratio of *A*_1_ does not exceed 60% until the room temperature is reached. It is suggested that the recombination in deep localized states shows better resistance against the non-radiative process in that the higher activation energy is needed for the delocalization process [51]. The resultant denotes that a majority of carriers in QWs prefer to recombine in the deep localized states, which is beneficial for higher radiation efficiency. Furthermore, the previous mentioned that V-shaped pits effectively block carriers from entering non-radiative recombination centers, also supports the reason that ratio of *A*_1_ remains below 60%.

### Device performance of InGaN-based red micro-LED

The next step involved fabricating a micro-LED device from the InGaN-based epitaxial structure. The fabrication process flow began with depositing indium tin oxide (ITO) as the current spreading layer on the top of the epitaxial structure. The 25 μm × 6 micro-LED mesa array were fabricated using photolithographic, and the ITO layer and mesa were etched by using both wet and dry etching. After that the samples underwent annealing to form a *p*-type ohmic contact, followed by the deposition of Ti/Al/Ti/Au metals as electrodes. The Al_2_O_3_ layer was deposited on the epi-wafer via atomic layer deposition (ALD), followed by SiO_2_ layer deposition through plasma-enhanced chemical vapor deposition (PECVD) and via hole process by inductively coupled plasma reactive ion etching (ICP-RIE). The passivation layer helps to repair surface damage and isolates the metal electrodes. A pad metal comprising Ti/Al/Au metals was evaporated, and finally, a distributed Bragg reflector (DBR) consisting of 5.5 pairs of SiO_2_/TiO_2_ layers was introduced to enhance the light extraction efficiency of packaged devices. For a more detailed description of the process and device cross-section diagram, refer to our previous work [30].

**Fig. 8:**
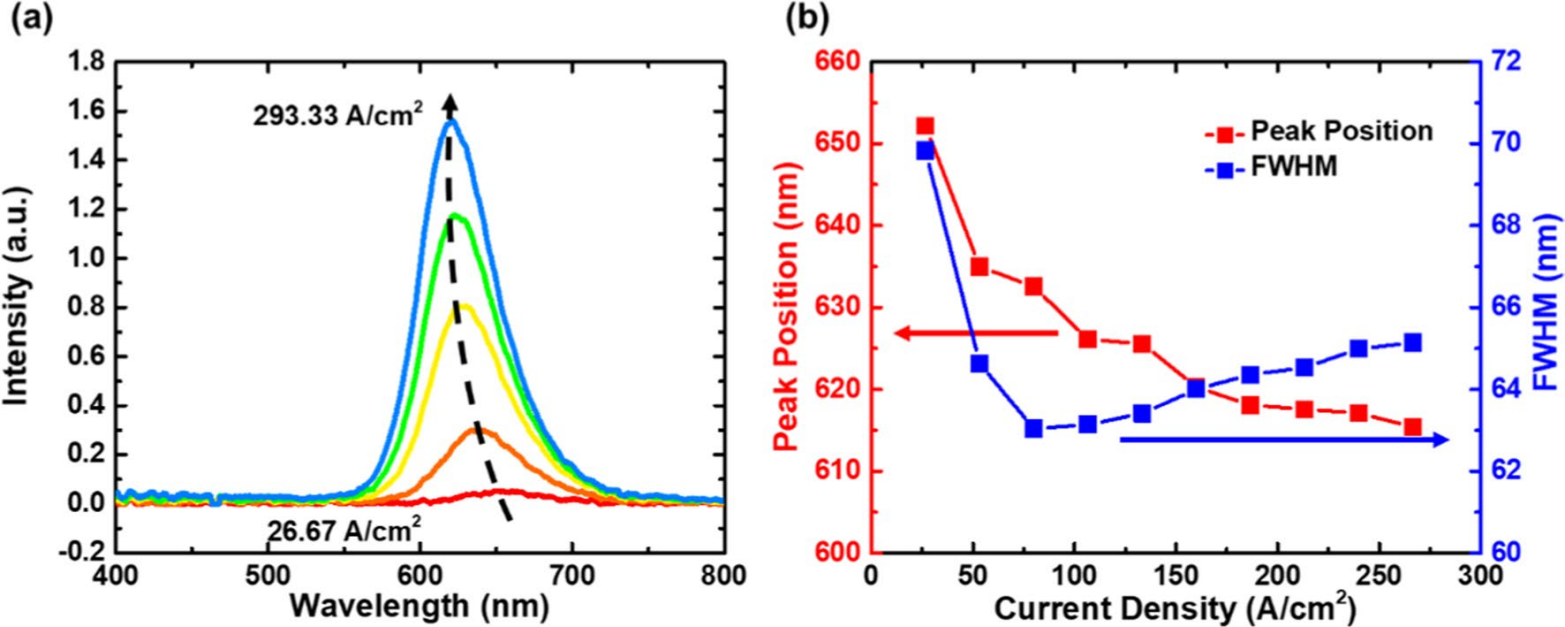
**a** Electroluminescence spectra at various current densities; **b** wavelength shift and FWHM as a function of current densities for the micro-LED device

The electroluminescence (EL) spectra of package LEDs at room temperature are summarized and shown in Fig. 8. The absence of blue emission in the EL spectra is due to the lower ability of hole to inject into n-side QWs [31] and the introduction of DBR for enhancing red emission extraction, thereby, lowering the additional blue emission. Though the peak position of EL spectra of InGaN-based red LED blueshift with respect to its PL spectra due to the variable junction temperature [58], the EL spectra exhibit similar behavior to the PL spectra, with a blueshift in peak position observed as the excitation source gets stronger. The peak position and FWHM of the red micro-LED device as a function of injected current density are shown in Fig. 8b. Both of them show the same trend as the power-dependent PL spectra. The value of FWHM at high current density is approximately 65 nm, which is comparable to those with the same epitaxial structure [24, 30]. The peak wavelength shifts from 652 to 615 nm as the current density increases from 26.67 to 293.33 A/cm^2^. However, the blueshift seems to be more negligible at a current density higher than 200 A/cm^2^. This phenomenon can be possibly attributed to the effect of heat generation on the InGaN active region [59–61]. In general, the heat accumulation due to the non-radiative process might lead to the redshift of the peak wavelength and the broadening of FWHM. However, the blueshift from band-filling effect is still prevailing, thereby counteracting the effect of heat generation. Hence, the much more slightly blueshift and FWHM increment are observed at high current density.

Sections 2 and 3 demonstrate that the epitaxial structure has a high. In content, resulting in a longer emission wavelength and V-pits that impede non-radiative recombination. Moreover, at room temperature, carriers can recombine in the deep localized state, leading to high radiative efficiency despite the increasing influence of non-radiative recombination at higher temperatures. Through in-depth analysis of the structural and optical properties in the former section, the epitaxial crystal shows great promising potential to enhance emission efficiency with long-wavelength emission. When compared to a previous study, the EQE values of InGaN-based red micro-LED enhance from 1.6% [24] to 5.0% [30] with increasing the density of V-pits increases from 1.28 × 10^8^ to 3.23 × 10^8^ cm^−2^. Therefore, the InGaN-based red LED with V-shaped pits provides a deep insight for improving quantum efficiency, and the EQE value is higher than that reported in a recent paper [62]. The results in temperature-dependent TRPL also confirm that the epitaxial structure show promise for high radiation efficiency.

Even though the epitaxial structure demonstrates a higher EQE, there is still a long way to go with green and blue InGaN-based LEDs. There are some methods available to enhance the device’s performance. Li et al. [63] formed an epitaxial tunnel junction contact to improve current spreading and reduce optical loss. Zhuang et al. [64] demonstrated that fabricating micro-holes in the planar mesa would also be beneficial to the performance of InGaN-based red LED. Hence, by taking advantage of the outstanding epitaxial structure, it is possible to achieve a higher EQE by utilizing the methods mentioned above.

## Conclusion

In this study, we investigated the structural and optical characteristics of a red micro-LED based on rich-indium InGaN with a high density of V-shaped pits. By utilizing lower-temperature growth of the active layer, the density of V-shaped pits was enhanced up to 3.23 × 10^8^ cm^−2^. TRPL analysis was conducted to estimate the IQE of the In_0.34_Ga_0.66_N-based red LED at room temperature, which was found to be approximately 12.22%. Furthermore, at low temperatures, strong confinement energies prevented carrier transfer to localized states. As the temperature increased above 100 K, non-radiative recombination began to dominate the recombination process. The majority of carriers then recombined in deeply localized states, which enhanced the radiative efficiency. Therefore, we can obtain a higher efficiency of EQE in the high indium content red InGaN-based LED. The study shows that V-shaped pits can effectively passivate the non-radiative recombination centers of threading dislocations, leading to a significant improvement in luminous efficiency. This finding highlights the importance of reducing or passivating non-radiative recombination in rich indium InGaN MQWs. It is hoped that this experimental result will contribute to the promising development of high-efficiency InGaN red micro-LED devices.

## Data Availability

The data presented in this study are available from the corresponding author upon reasonable request.
